# Supplementary material for: A CRISPR toolbox for generating intersectional genetic mouse models for functional, molecular, and anatomical circuit mapping
Source: BMC Biol. 2022 Jan 28;20:28. doi: 10.1186/s12915-022-01227-0 (PMC8796356; doi:10.1186/s12915-022-01227-0)

Supplemental Figure 1

A Genetic schema for Figure 3 A-H

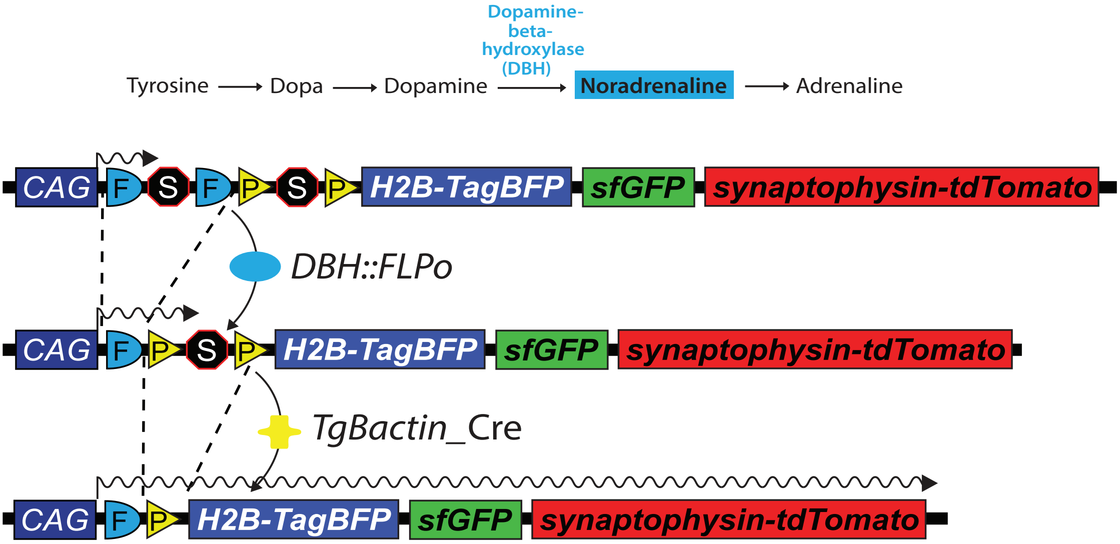

B Genetic schema for Figure 3 I-P

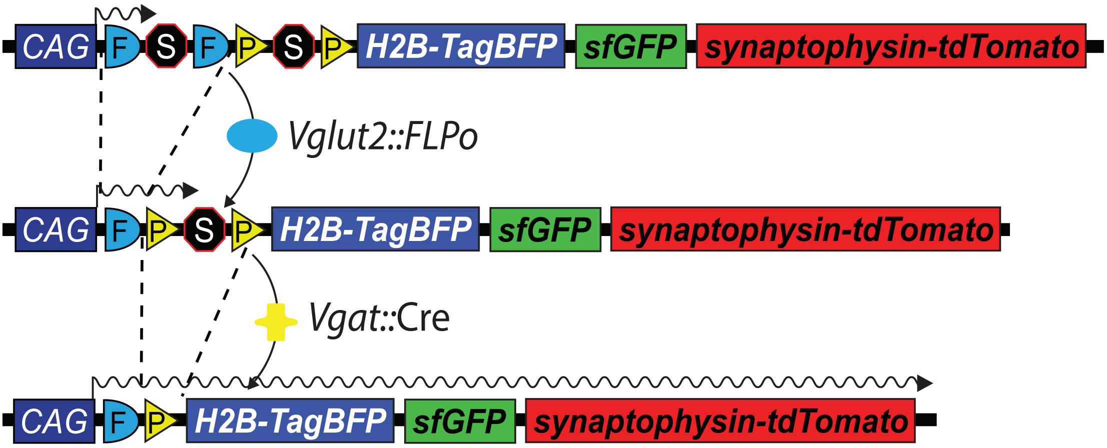

C Genetic schema for Figure 4

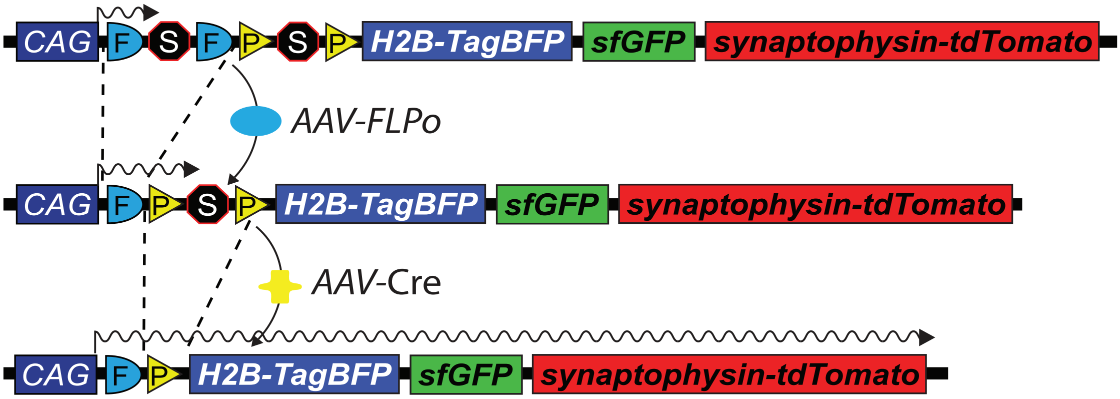

Supplement: Supplementary file 1 — Additional file 1: Figure S1. Genetic schema for animals used to gather data for Figures 3 and 4. Vector diagrams for the transgenes present in mice used for anatomical fluorescence experiments in Figures 3 and 4. Demonstration of source of recombinase for animals from each panel in the figures. [file 12915_2022_1227_MOESM1_ESM.pdf]
