# Supplementary figures and images for: A CRISPR toolbox for generating intersectional genetic mouse models for functional, molecular, and anatomical circuit mapping
Source: BMC Biol. 2022 Jan 28;20:28. doi: 10.1186/s12915-022-01227-0 (PMC8796356; doi:10.1186/s12915-022-01227-0)

# Supplemental Figure 2

Breeding schema for *RC::P\_DREADD*; *RC::ePe*; *TgDBH\_Cre*

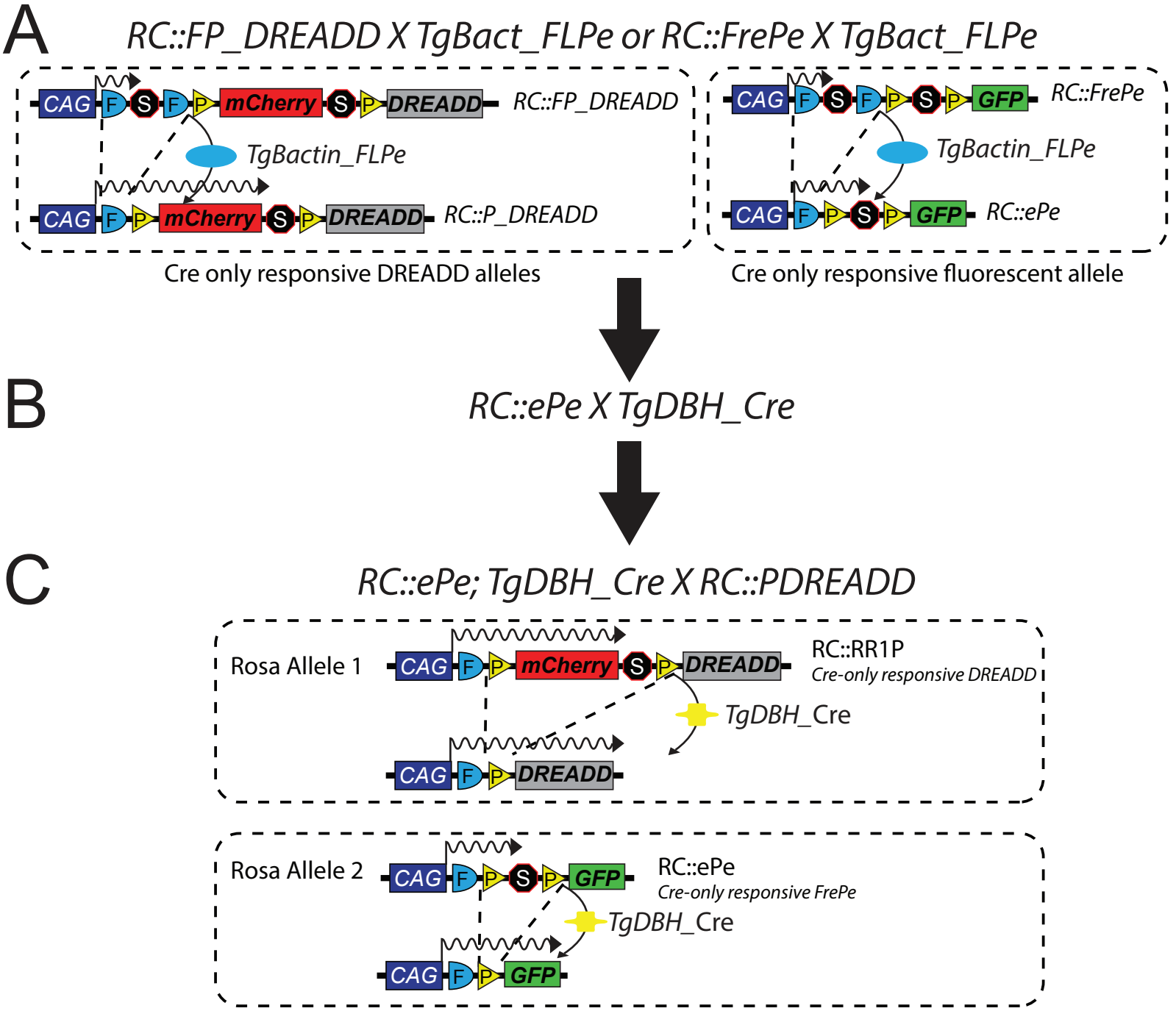

Supplement: Supplementary file 2 — Additional file 2: Figure S2. Breeding schema for animals used in Figures 7-9. Vector diagrams for animals used in e-physiology and whole animal physiology studies in Figures 7-9. Breeding paradigms indicated with arrows show recombinase and effector molecule source during derivation of used lines. [file 12915_2022_1227_MOESM2_ESM.pdf]
