# Supplementary material for: A CRISPR toolbox for generating intersectional genetic mouse models for functional, molecular, and anatomical circuit mapping
Source: BMC Biol. 2022 Jan 28;20:28. doi: 10.1186/s12915-022-01227-0 (PMC8796356; doi:10.1186/s12915-022-01227-0)

# Supplemental Figure 5

## QQplots for normal distribution testing of residuals from plethysmography analyses from Fig. 9, RR2 (hM3D).

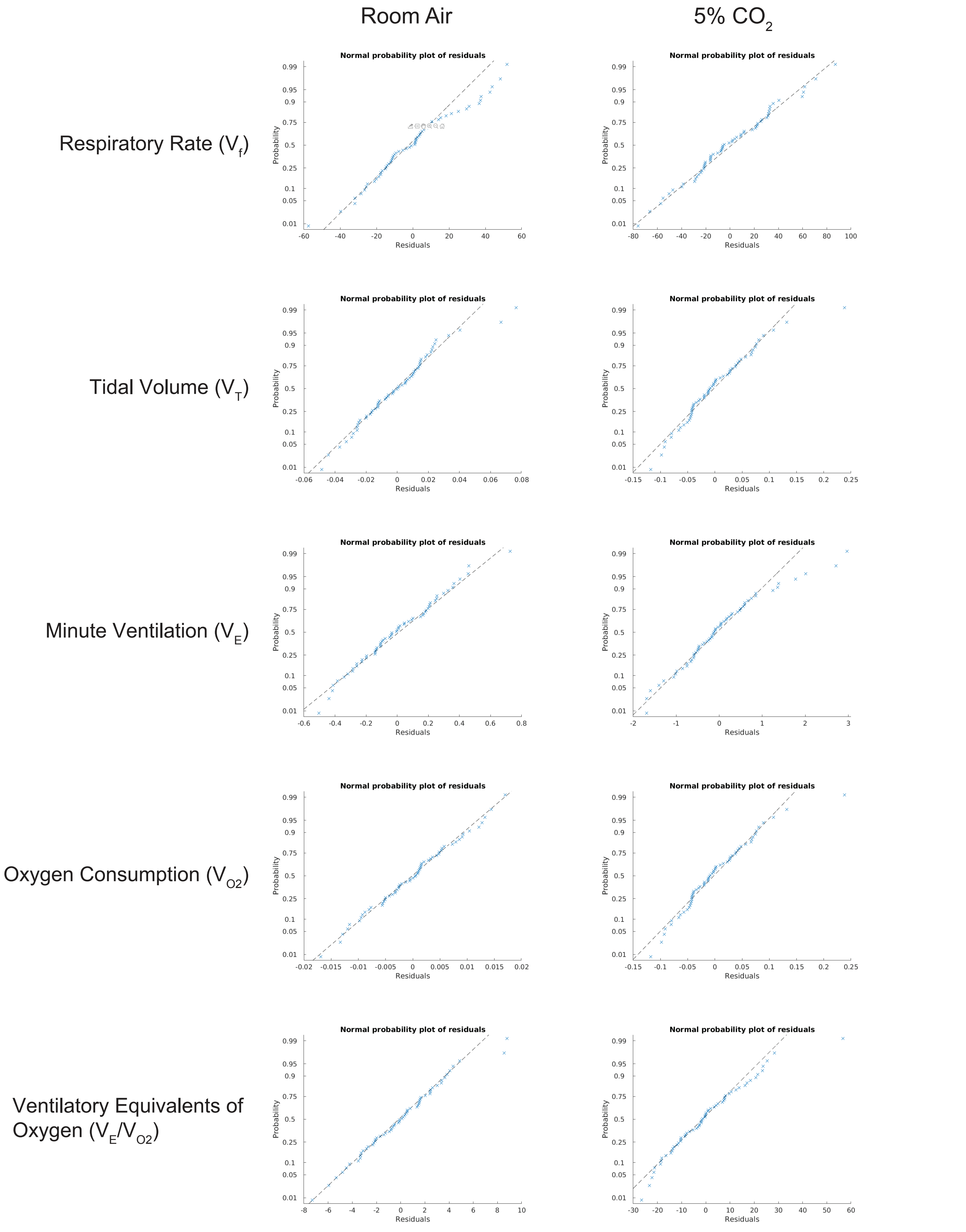

Supplement: Supplementary file 5 — Additional file 5: Figure S5. QQplots for normal distribution testing of residuals from plethysmography analyses from Fig. 9. Plot of the empirical quantiles of the residual distributions vs the theoretical quantiles of gaussian distributions. [file 12915_2022_1227_MOESM5_ESM.pdf]
