# Supplementary material for: A CRISPR toolbox for generating intersectional genetic mouse models for functional, molecular, and anatomical circuit mapping
Source: BMC Biol. 2022 Jan 28;20:28. doi: 10.1186/s12915-022-01227-0 (PMC8796356; doi:10.1186/s12915-022-01227-0)

Supplemental Figure 6

Residual plots for independence testing of residuals from plethysmography analyses from Fig. 9, RR2 (hM3D).

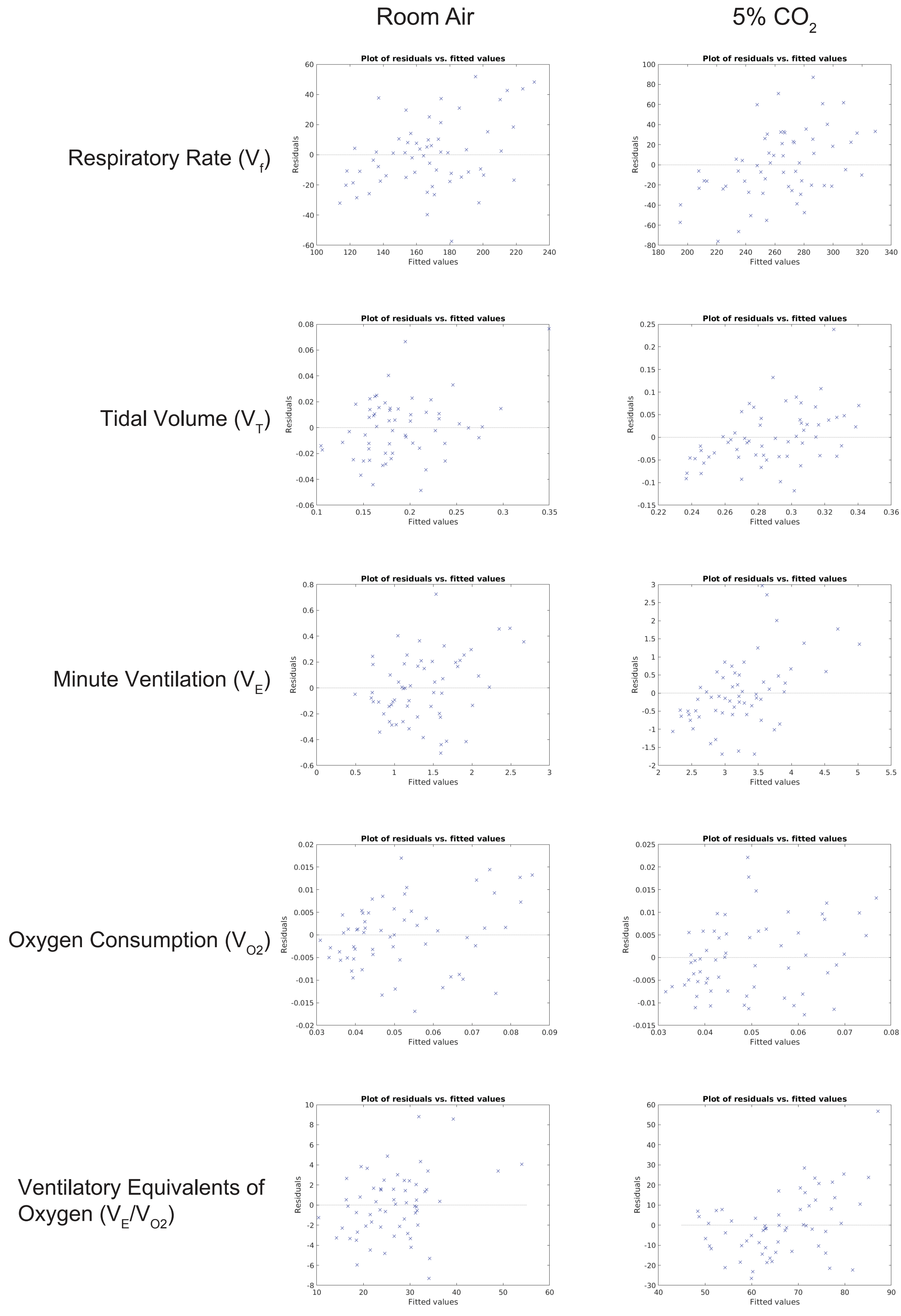

Supplement: Supplementary file 6 — Additional file 6: Figure S6. Residual plots for independence testing of residuals from plethysmography analyses from Fig. 9. Description of data: Plot of residuals vs model predicted values of the dependent variable. [file 12915_2022_1227_MOESM6_ESM.pdf]
